# Thrombin Generation Capacity of Prothrombin Complex Concentrate in an *In Vitro* Dilutional Model

**DOI:** 10.1371/journal.pone.0064100

**Published:** 2013-05-17

**Authors:** Oliver Grottke, Rolf Rossaint, Yvonne Henskens, Rene van Oerle, Hugo ten Cate, Henri M. H. Spronk

**Affiliations:** 1 Department of Anesthesiology, RWTH Aachen University Hospital, Aachen, Germany; 2 Central Diagnostic Laboratory, Maastricht University Medical Center, Maastricht, The Netherlands; 3 Laboratory for Clinical Thrombosis and Haemostasis, Department of Internal Medicine, Cardiovascular Research Institute Maastricht, Maastricht University Medical Center, Maastricht, The Netherlands; Institut National de la Santé et de la Recherche Médicale, France

## Abstract

**Background:**

The use of PCC for the treatment of trauma-induced coagulopathy potentially increase the risk of thromboembolism and disseminated intravascular coagulation, which is addressed to an imbalance of both pro- and anticoagulants. As PCCs differ in composition, we used an in vitro dilutional approach to assess the overall thrombin generation of five different PCCs through various laboratory assays.

**Methods:**

The vitamin K-dependent coagulation factors, heparin, and antithrombin were assessed in five commercially available PCCs. The procoagulant potential of the PCCs was assessed in plasma and whole blood from 4 healthy donors by means of classical coagulation assays, thrombin generation assay and thromboelastometry. In order to reflect coagulopathy, whole blood was diluted to 80, 60, 40, and 20% with Ringer’s lactate solution.

**Results:**

The five different PCCs were characterised by comparable levels of factors II, VII, IX and X (all around 20–30 IU/mL), whereas the heparin (0 to 17.6 IU/mL) and antithrombin (0.06 to 1.29 IU/mL) levels were remarkably different between manufactures. *In vitro* dilution of blood induced a prolongation of the PT and aPTT, and attenuation of thrombin generation and ExTem induced thromboelastometry. Overall, non- or low-heparin containing PCCs restored the *in vitro* dilutional coagulopathy, whereas PCCs containing heparin have an anticoagulant effect. The thrombin generation assay showed to be the most sensitive method for assessment of PCC effects.

**Conclusions:**

This study shows that most available PCCs are not balanced regarding their pro- and anticoagulants. The effect of measured differences in thrombin generation among different PCCs requires further investigations to elaborate the clinical meaning of this finding in the treatment of trauma induced coagulopathy.

## Introduction

Trauma induced coagulopathy (TIC) in severely injured patients is a complex bleeding disorder contributing to exsanguination and to a high mortality in the early phase after hospitalization [1]. To protect trauma victims from massive blood loss and secondary systemic complications, early termination of TIC is essential. Although most trauma centers use fresh frozen plasma (FFP) for restoration of coagulation factors, this therapy has several drawbacks, including transfusion-related acute lung injury (TRALI), volume overload, and allergic reactions [2]–[4]. Studies over the past years have investigated the impact of specific coagulation factors concentrates, such as prothrombin complex concentrates (PCCs) and fibrinogen to terminate hemorrhages and to reinstate hemostasis [5]–[12]. In contrast to FFP, coagulation factor concentrates are immediately available facilitating early and prompt correction of coagulopathy while avoiding further dilution of coagulation factors.

PCCs are derived from human plasma and contain the vitamin K-dependent coagulation factors prothrombin (fII), factor VII (fVII), fIX, and fX. Both PCCs with low levels of fVII (3-factor PCCs, commonly used in the US) and higher concentrations of fVII (4-factor PCCs, applied mainly in the EU) are available. The composition of PCCs differs considerably between manufacturers and the products may contain the anticoagulants protein C, protein S, antithrombin, as well as heparin [13]. Generally, PCCs are commonly used for a rapid reversal of oral anticoagulation (vitamin K antagonists) and treatment of individuals with a deficiency of vitamin K-dependent coagulation factors, such as in patients with liver failure or life-threatening bleeding [14], [15]. For the reversal of oral anticoagulation several studies have been performed and the efficacy and safety for this indication seems overall acceptable [16]. In contrast, all evidence supporting the use of PCCs in trauma-related coagulopathy is from animal and human observational studies [5]–[12], [17].

The primary safety concerns with the use of PCCs are thromboembolic complications either due to overdosing or to excessive concentrations of prothrombin in relation to the levels of anticoagulatory proteins [18]. However, PCCs are standardized and administered based on the quantity of fIX and alterations in the ratio between pro- and anticoagulant factor levels between PCCs may add to differences in thrombin generating potential. In a recent animal trial employing a model of hemodilution and trauma induced coagulopathy it was shown that administration of a high dose of PCC induced thromboembolism and increased the risk of disseminated intravascular coagulation (DIC [17]. As this study investigated only one brand of PCC, no conclusions about the thrombin generation capacity of other available PCC preparations can be drawn.

Laboratory measurements to assess thrombin generation are available and have been proposed for guiding PCC administration [16]. However, none of these assays have been applied for this purpose in bleeding trauma patients.

To assess the overall thrombin generating capacity of PCCs, we investigated the overall procoagulant potential of five different PCCs in an in vitro whole blood dilutional setting. The impact on coagulation was assessed by global coagulation tests, rotational thromboelastometry and thrombin generation measurements using the Calibrated Automated Thrombogram (CAT).

## Materials and Methods

### Prothrombin Complex Concentrates

The following PCCs were applied: CoFact 500 I.E. (Sanquin, Amsterdam, The Netherlands; lot 10D29G321A), Beriplex P/N 250 (CSL Behring GmbH, Marburg, Germany; lot 56560111C), Prothromplex NF 600 (Baxter, Vienna, Austria; lot VNP5L003), Octaplex 500 (Octapharma, Vienna, Austria; lot A122C261A), and PPSB-human SD/Nano 600 (Octapharma Vienna, Austria; lot E137A262). Prior to testing, the lyophilised PCCs were reconstituted with sterile water according to the manufacturers instructions. Aliquots were stored at −80°C and thawed at −37°C for 10 min prior to application.

### Healthy Donors, Blood Collection

Written informed consent was obtained from all participants, and the study was approved by the local Medical Ethical Review Board azM/UM. Venous blood was collected from four healthy volunteers through antecubital venipuncture using 21-gauge needles, one discarding tube and four consecutive 3.2%(w/v) citrated Vacutainer glass tubes (Becton Dickinson, Plymouth, UK). Whole blood from each donor was pooled and for the global coagulation and thrombin generation assays, whole blood was diluted to 80, 60, 40, and 20% with Ringer’s lactate (RL, Fresenius, Bad Homburg, Germany) solution containing 3.2%(w/v) citrate. Undiluted and diluted whole blood aliquots were supplemented with one of the five PCC preparations to obtain 0.53 IU fIX/mL. This dose was based on the 35 IU PCC per kg body weight, as previously described and an average human weight of 85 kg with a total blood volume of 5.6 L [16]. 0.9%(w/v) saline solution was added as control. A subset was supplemented with fibrinogen (Haemocomplettan P, CSL Behring, Germany) to correct for the dilutional effect and to obtain a final fibrinogen concentration comparable to that of undiluted whole blood (100%). All blood dilution tubes were centrifuged for 5 min at 2,000×g, followed by a second step at 10,000×g for 10 min. Aliquots of (diluted) plasma were snap frozen in liquid nitrogen and stored at −80°C until analysis. For thromboelastometry, whole blood was diluted to 70, and 40% with RL solution containing 3.2%(w/v) citrate directly after collection followed by addition of 0.9%(w/v) saline solution or each of the five PCCs at a dose of 0.53 UI fIX/mL. Again, a subset was supplemented with fibrinogen to reach the baseline values of undiluted whole blood (100%).

### Global Coagulation Assays

All coagulation assays were performed according to the manufacturers instructions. Factors II, VII, and X were assessed through a one-stage clotting assay using reagents TromborelS (Siemens, Marburg, Germany), corresponding factor deficient plasmas (Siemens) and SHP (Siemens) as calibrator. Factor IX was assessed by a one-stage clotting assay with ActinFS (Siemens) as activator and SHP (Siemens) as calibrator and the corresponding factor IX-deficient plasma (Siemens). Antithrombin levels were measured by the Berichrom Antithrombin III assay, protein C:act by the Berichrom Protein C Assay (Siemens), free protein S by the HemosIL Free Protein S assay (Instrumentation Laboratory, Breda, The Netherlands), heparin was assessed by means of the anti Xa levels using the BIOPHEN Heparin Anti-Xa (Hyhen Biomed, Nueville-sur-Oise, France), the prothrombin time (PT) by Innovin (Siemens) reagent, the aPTT by ActinFSL reagent (Siemens), and fibrinogen through the Thrombin (Siemens) reagent. All analysis were performed on a CA-7000 Sysmex (Siemens) system.

### Thrombin Generation

Thrombin generation in human platelet-poor plasma was measured by means of the Calibrated Automated Thrombogram method (Thrombinoscope BV, Maastricht, The Netherlands), which employs a low affinity fluorogenic substrate for thrombin (Z-Gly-Gly-Arg-AMC) to continuously monitor thrombin activity in clotting plasma. Measurements were conducted on 80 µL human PPP in a total volume of 120 µL (20 µL fluorogenic substrate, Calcium chloride (FluCa) and 20 µL trigger reagent containing tissue factor (TF) and phospholipids (PL)). The following experimental conditions were used: 0 pM TF with 4 µM PL at 20∶20∶60 mol% PS:PE:PC, 1 pM TF with 4 µM PL, and 5 pM TF with 4 µM PL. In order to correct for inner-filter effects and substrate consumption, each thrombin generation measurement was calibrated against the fluorescence curve obtained in a sample from the same plasma (80 µL), added with a fixed amount of thrombin-α2-macroglobulin complex (20 µL Thrombin Calibrator, Thrombinoscope BV) and 20 µL FluCa. Fluorescence was read in a Fluoroskan Ascent reader (Thermo Labsystems OY, Helsinki, Finland) equipped with a 390/460 filter set and thrombin generation curves were calculated with the Thrombinoscope software (Thrombinoscope BV).

Rotational thromboelastometry (ROTEM, TEM International, Munich, Germany) analysis was performed using the following standard assays according to the manufacturer’s recommendations at 37°C: InTem (partial thromboplastin and ellagic acid) and ExTem (tissue factor). The following parameters were obtained: clotting time (CT in seconds), maximum clot firmness (MCF in mm), and the alpha angle in degrees. All measurements were performed in duplicate.

### Statistical Analysis

For graphical purposes and statistical analysis, GraphPad Prism (Version 5.01; GraphPad Software Inc, La Jolla, CA) was used. Data are presented as median with inter-quartile ranges (IQRs) or median with range (min-max) based on duplicate measurements from four healthy individuals. Differences between undiluted and 80, 60, 40, and 20% dilution with RL solution were analyzed for significance by means of the Friedman Test (ANOVA for repeated measurement and nonparametric distribution) with a two-tailed significance level of alpha equals 0.05. Differences between groups were assessed through Mann-Whitney test with a two-tailed significance level of alpha equals 0.05. For the clearness of data presentation not all significant differences are displayed.

## Results

### Composition of PCCs

The coagulation factor activities of the single lots measured for the five different PCCs are provided in Table 1. In comparison to the ranges declared by the manufactures, assessed coagulation factors prothrombin, fVII, and fX were all within the given ranges and varied between 26 and 37 IU/mL. Analyzed factor IX quantities were below the manufactures specifications in three out of five PCCs. Remarkably, the levels of anticoagulants protein C, protein S, antithrombin and heparin were not always provided by the manufactures, although all five different PCCs contained these constituents. Proteins C and S levels were comparable between all PCCs, whereas only Cofact 500 did not contain heparin. Prothromplex NF 600, Octaplex 500 and PPSB-human SD/Nano 600 contained up to 18 IU/mL of heparin.

**Table 1:** Coagulation factor activities of the single lots measured for the five different PCCs.

PCC		FII	FVII	FIX	FX	Protein C	Free Prot S	AT	Anti Xa
		IU/mL	IU/mL	IU/mL	IU/mL	IU/mL	IU/mL	IU/mL	IU/mL
**CoFact**	**Insert**	14–35	7–20	25	14–35	no value	no value	no value	no value
	**Measured**	**29**	**21**	**22**	**23**	**19**	**14**	**0.26**	**<0.05**
**Beriplex**	**Insert**	20–48	10–25	20–31	22–45	15–45	no value	0.2–1.5	0.4–2
	**Measured**	**26**	**16**	**23**	**28**	**23**	**27**	**0.56**	**0.7**
**Prothromplex**	**Insert**	30	25	30	30	no value	no value	no value	no value
	**Measured**	**33**	**27**	**20**	**27**	**26**	**26**	**1.25**	**14.4**
**OctaPlex**	**Insert**	14–38	9–24	25	18–30	13–31	12–32	no value	5–12.5
	**Measured**	**26**	**19**	**20**	**23**	**19**	**21**	**0.06**	**17.6**
**Nano 600**	**Insert**	25–55	7.5–20	24–37.5	25–55	20–50	5–25	present	present
	**Measured**	**37**	**18**	**22**	**33**	**23**	**18**	**1.29**	**6.6**

The first row indicates the values as provided by the manufacturer (Insert) and the second row the values as measured in house (Measured). The levels of heparin are presented as AntiXa levels in IU/mL. “no value” indicates that the presence or level of this factor was not provided by the manufacturer.

### Whole Blood Dilution, Fibrinogen Substitution and Haemostasis

**Figure 1:**
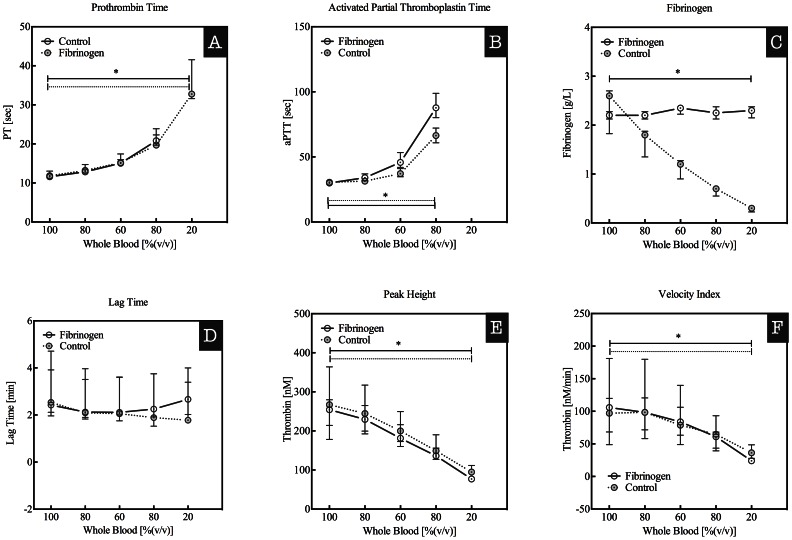
Prothrombin times (PT in sec, panel A), activated partial thromboplastin times (aPTT in sec, panel B), fibrinogen levels (in g/L, panel C), as well as the plasma thrombin generation characteristics lag time (in min, panel D), peak height (in nM thrombin, panel E), and velocity index (in nM/min, panel F) for plasmas derived from whole blood diluted with Ringers Lactate (RL) to obtain undiluted and 80, 60, 40, and 20% whole blood. Solid lines indicate control blood without addition of fibrinogen and dashed lines indicate diluted whole blood to which fibrinogen was added in order to obtain a final concentration of approximately 2.5 g/L. Data are presented as median with IQR, *indicates p<0.05.

Whole blood was diluted in Ringer’s lactate (RL) solution containing 3.2%(w/v) citrate to obtain undiluted and 80, 60, 40, and 20% diluted blood. Citrate was added in order to prevent coagulation. Dilution of whole blood from 100 to 40% caused a prolongation of the PT from 11.6±0.5 sec to 20.9±1.4 sec, a prolongation of the aPTT from 30.8±1.5 sec to 66.5±6.0 sec (p<0.05), and a decrease in fibrinogen levels from 2.4±0.5 g/L to 0.7±0.1 g/L (p<0.05) (Figure 1: panels A, B, and C). At 20% whole blood the PT and aPTT were prolonged beyond the upper detection limit, whereas the fibrinogen levels dropped further to 0.3±0.05 g/L. Substitution of fibrinogen levels to approximately the values at undiluted whole blood (100%) (approximately 2.5 g/L) had little to no effect on the length of the PT and aPTT upon dilution of whole blood (Figure 1: panels A, B, and C).

*In vitro* thrombin generation, triggered with 5 pM tissue factor, was characterized by a lag time of 3.12±1.51 min, a peak height of 270±97 nM and a velocity index of 109±70 nM/min in plasma from undiluted whole blood. The lag time gradually shortened to 2.28±1.08 min at 20% whole blood, accompanied by an attenuation of the peak height to 98±12 nM and velocity index to 37±11 nM/min (both p<0.05; Figure 1: panels D, E, and F). Addition of fibrinogen to diluted whole blood caused a comparable lag time for all dilutions, whereas the peak height and velocity index decreased from 249±34 nM to 77±3 nM and from 98±28 nM/min to 24±1 nM/min (both p<0.05), respectively (Figure 1: panels D, E, and F). Nevertheless, thrombin generation in plasma from diluted whole blood was comparable to that of diluted whole blood supplemented with fibrinogen to obtain baseline values.

**Figure 2:**
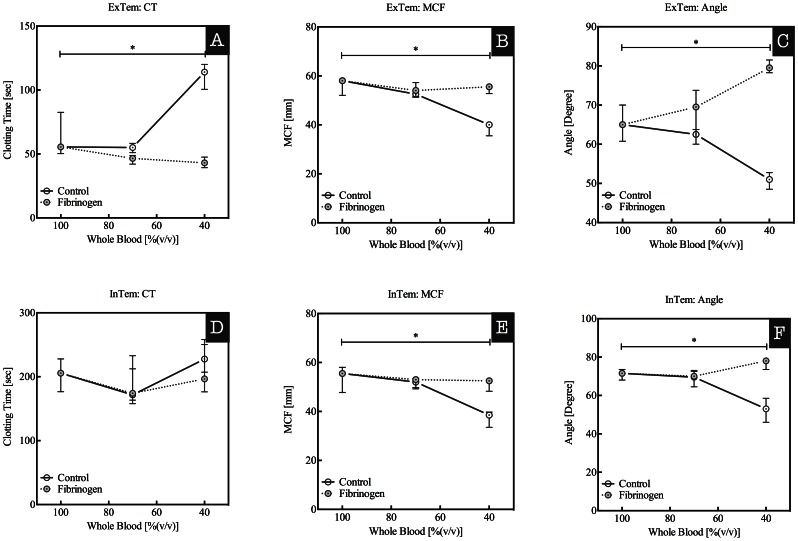
ExTem (upper panel) and InTem (lower panel) derived thromboelastometry (ROTEM) parameters from whole blood diluted with Ringers Lactate (RL) to obtain undiluted and 80, 60, 40, and 20% whole blood. Solid lines indicate control blood without addition of fibrinogen and dashed lines indicate diluted whole blood to which fibrinogen was added in order to obtain a final concentration of approximately 2.5 g/L. Panel A and D: Clotting times (CT in sec), panel B and E: maximum clot firmness (MCF in mm), and panel C and F: alfa-angle (in degrees). Data are presented as median with IQR, *indicates p<0.05.

Extrinsic pathway triggered ROTEM baseline analysis, ExTem, showed a CT of 63±19 sec, a maximum clot firmness MCF of 56±4 mm, and a α-angle of 71±3 degree. Dilution of whole blood to 70% did not influence the ROTEM analysis significantly as compared to baseline values. However, dilution of whole blood to 40% induces a prolongation of the CT to 111±11 sec and reduction of the MCF to 39±3 mm and of the α-angle to 51±2 degree (Figure 2: panels A, B, and C; 100% vs. 40% p<0.05). Addition of fibrinogen to 40% diluted whole blood samples restored the CT and MCF values to 43±4 sec and 55±2 mm. The α-angle value was restored beyond the baseline value to 80±2 degree. Dilution of whole blood did not change the CT of intrinsic activated ROTEM analysis: 203±28 sec at baseline compared to 228±24 sec at 40% (Figure 2, panels D, E, and F). The MCF decreased from 54±6 mm to 37±4 mm and the α-angle from 71±3 to 53±7 degree (both p<0.05). InTem CT, MCF, and α-angle values of 40% whole blood in the presence of baseline fibrinogen concentrations were comparable to those obtained for whole blood at baseline (CT: 203±28 sec vs. 210±45 sec, MCF: 54±6 mm vs. 51±3 mm, α-angle: 71±3 degree vs. 77±3 degree).

### Addition of PCC

**Figure 3:**
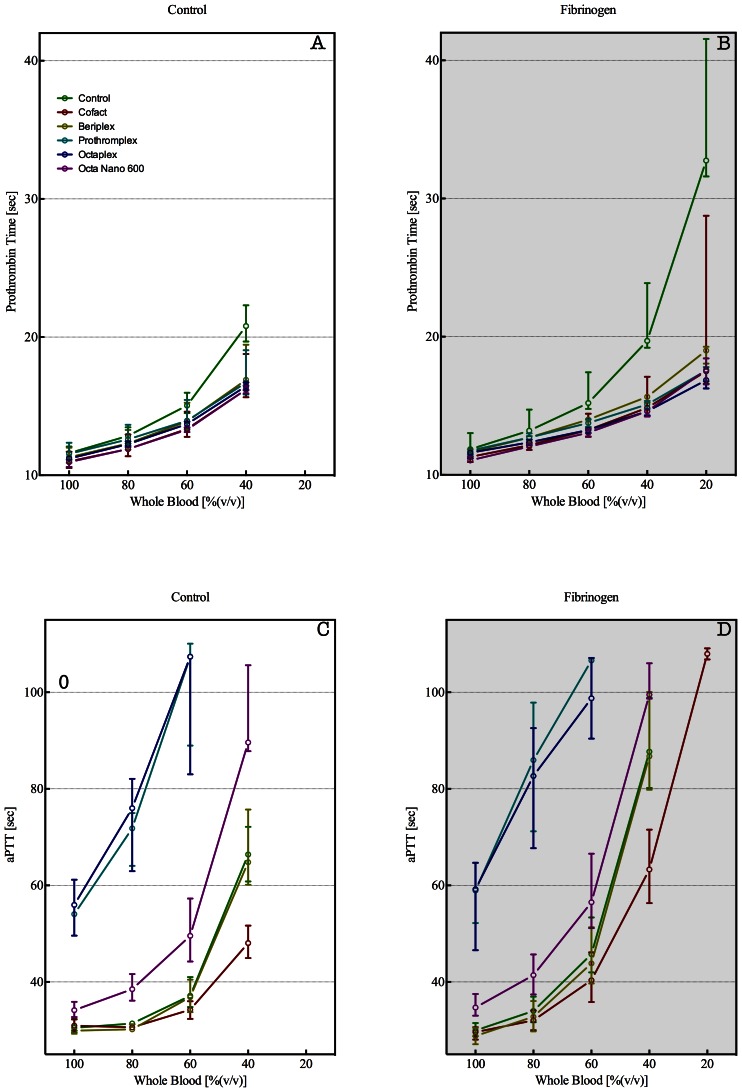
Prothrombin times (PT in sec, panel A and B) and activated partial thromboplastin times (aPTT in sec, panel C and D) for plasmas derived from whole blood diluted with Ringers Lactate (RL) to obtain undiluted and 80, 60, 40, and 20% whole blood. Before preparation of plasma, PCCs were added to undiluted (100%) and diluted (80, 60, 40, and 20%) whole blood (panels A and C). To correct for fibrinogen dilution, whole blood was supplemented with fibrinogen in order to obtain a final concentration of approximately 2.5 g/L at each dilution (panels B and D). Control (green), CoFact 500 I.E. (red), Beriplex P/N 250 (gold), Prothromplex NF 600 (aqua), Octaplex 500 (blue), and PPSB-human SD/Nano 600 (purple). Data are presented as mean with SEM.

Adding PCCs to whole blood (100%) did not alter the PT, whereas addition to diluted whole blood shortened the PT for all PCCs tested at all dilutions, with the most clear effect at 40 and 20% blood (Figure 3A). At the latter dilution, the PT was prolonged above the upper limit of detection in both the absence and presence of PCC, whereas addition of PCCs at constant fibrinogen levels shortened the PT (Figure 3B). At 40% whole blood, addition of PCC shortened the PT and co-application with fibrinogen caused a further shortening of the PT. The aPTT showed a clear distinction between heparin containing PCCs (Prothromplex NF 600, Octaplex 500 and PPSB-human SD/Nano 600) and those without heparin (Cofact) or low heparin levels (Beriplex). Addition of heparin containing PCCs caused a prolongation of the aPTT in undiluted and diluted whole blood, whereas addition of non-heparin or low-heparin PCCs did not alter the aPTT (Figure 3). Remarkably, Prothromplex NF 600 and Octaplex 500 prolonged the aPTT beyond the upper detection limit at 40 and 20% whole blood with RL.

**Figure 4:**
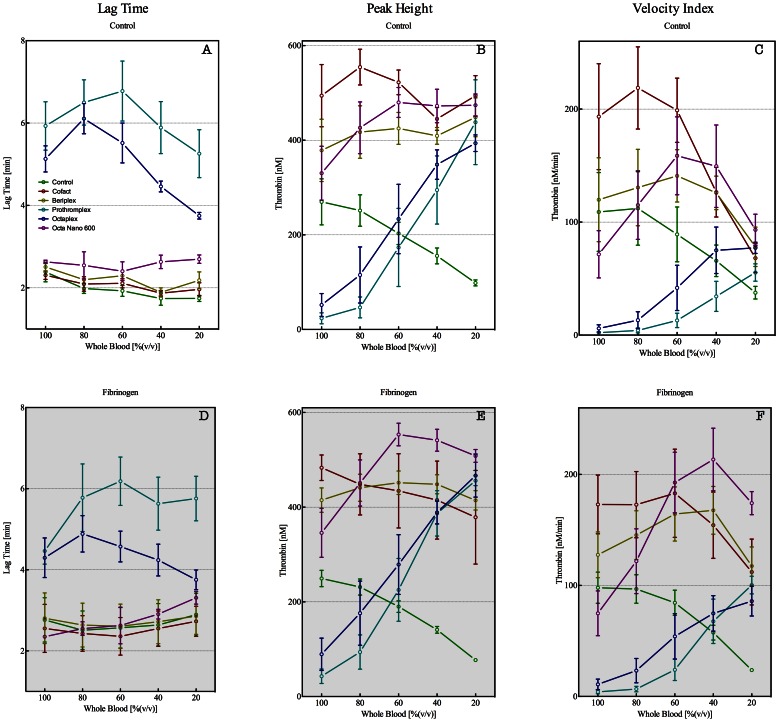
Plasma thrombin generation, triggered with 5 pM tissue factor, parameters lag time (in min, panel A and D), peak height (in nM thrombin, panel B and E), and velocity index (in nM/min, panel C and F) for plasmas derived from whole blood (100%) diluted with Ringers Lactate (RL) to obtain 80, 60, 40, and 20% whole blood. Before preparation of plasma, PCCs were added to undiluted (100%) and diluted (80, 60, 40, and 20%) whole blood (panels A, B, and C). To correct for fibrinogen dilution, whole blood was supplemented with fibrinogen in order to obtain a final concentration of approximately 2.5 g/L at each dilution (panels D, E, and F). Control (green), CoFact 500 I.E. (red), Beriplex P/N 250 (gold), Prothromplex NF 600 (aqua), Octaplex 500 (blue), and PPSB-human SD/Nano 600 (purple). Data are presented as mean with SEM.

In general, non-heparin containing PCCs enhanced thrombin generation as indicated by elevation of the peak height and velocity index. In plasma from undiluted blood, the contribution of heparin-free PCCs was rather marginal, but at higher dilution (40% blood) thrombin generation was significantly increased by Cofact (445±48 nM), Beriplex (409±36 nM) and PPSB-human SD/Nano 600 (472±71 nM, Figure 4 all p<0.05) as compared to control (156±34 nM). The heparin containing PCCs, however, caused inhibition of thrombin generation in plasma from undiluted whole blood, as well as from 80 and 60% whole blood. Overall, our data suggests that plasma thrombin generation, triggered with 5 pM tissue factor, could distinguish between the presence or absence of heparin in the applied PCCs. Addition of Prothromplex NF 600 and Octaplex 500 to undiluted whole blood caused an attenuation of the peak height from 270±97 nM to 24±23 nM and 52±48 nM, respectively (all p<0.05). This anticoagulant effect of the heparin containing PCCs was diminished at higher dilution of the whole blood, eg. 40 and 20% whole blood. At these higher dilutions, addition of Prothromplex NF 600 and Octaplex 500 induced an enhanced thrombin generation compared to control plasma. In general, addition of fibrinogen had no major effects on the outcome of thrombin generation data (data not shown).

**Figure 5:**
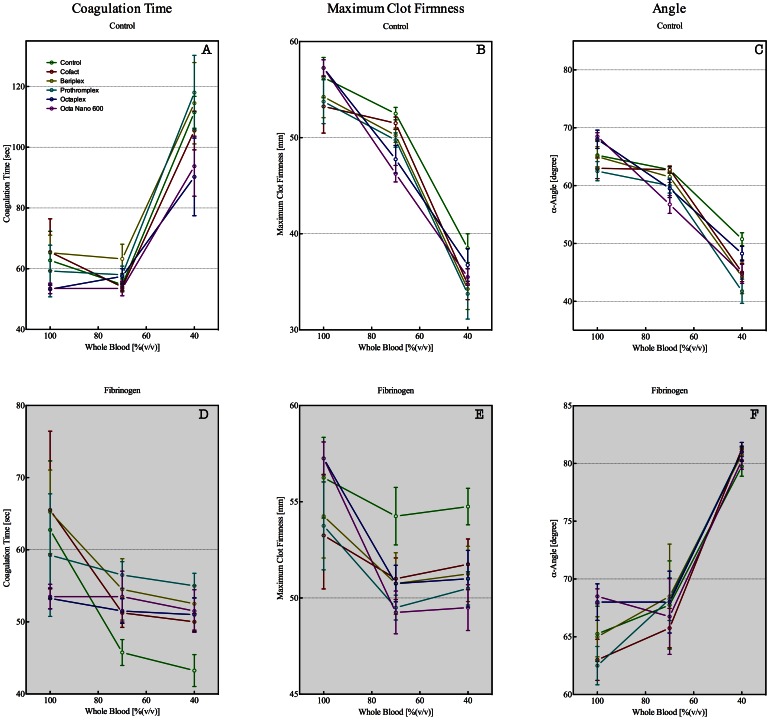
Thromboelastometry (ROTEM), triggered with ExTem reagent, parameters coagulation time (CT in sec, panel A and D), maximum clot firmness (MCF in mm, panel B and E), and α-angle (in degrees, panel C and F) for samples derived from whole blood (100%) diluted with Ringers Lactate (RL) to obtain 70, and 40% whole blood. PCCs were added to undiluted (100%) and diluted (70 and 40%) whole blood. To correct for fibrinogen dilution, whole blood was supplemented with fibrinogen in order to obtain a final concentration of approximately 2.5 g/L at each dilution (panels D, E, and F). Control (green), CoFact 500 I.E. (red), Beriplex P/N 250 (gold), Prothromplex NF 600 (aqua), Octaplex 500 (blue), and PPSB-human SD/Nano 600 (purple). Data are presented as median with IQR.

**Figure 6:**
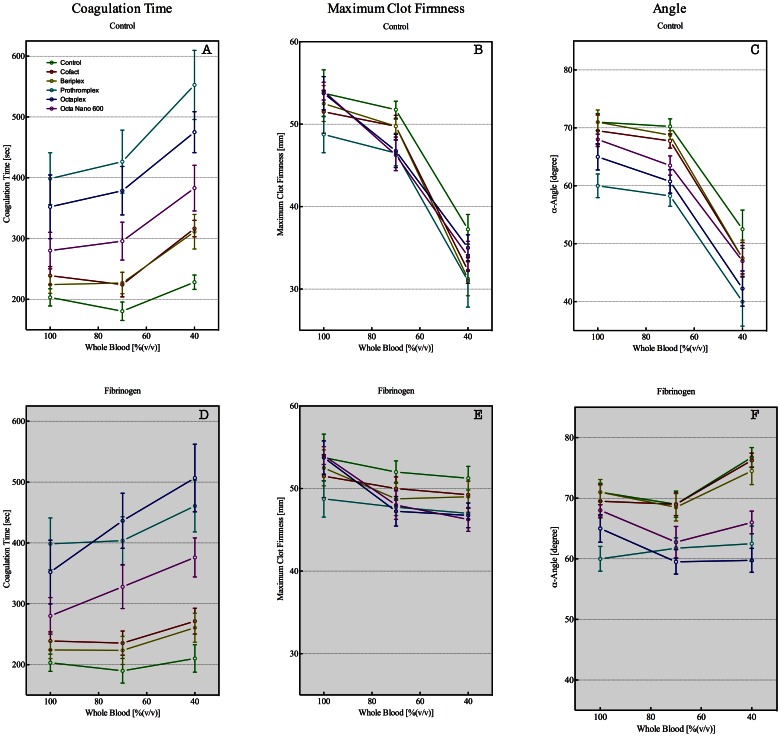
Thromboelastometry (ROTEM), triggered with InTem reagent, parameters coagulation time (CT in sec, panel A and D), maximum clot firmness (MCF in mm, panel B and E), and α-angle (in degrees, panel C and F) for samples derived from whole blood (100%) diluted with Ringers Lactate (RL) to obtain 70, and 40% whole blood. PCCs were added to undiluted (100%) and diluted (70 and 40%) whole blood. To correct for fibrinogen dilution, whole blood was supplemented with fibrinogen in order to obtain a final concentration of approximately 2.5 g/L at each dilution (panels D, E, and F). Control (green), CoFact 500 I.E. (red), Beriplex P/N 250 (gold), Prothromplex NF 600 (aqua), Octaplex 500 (blue), and PPSB-human SD/Nano 600 (purple). Data are presented as median with IQR.

Addition of PCCs to diluted whole blood (at 70 and 40% whole blood) did not recover the coagulopathy effects observed in ExTem and InTem ROTEM analysis (Figures 5 and 6). Both the ExTem and InTem ROTEM analysis for diluted whole blood were comparable between controls and the non-heparin containing PCCs (Cofact and Beriplex), whereas the heparin containing PCCs caused a prolongation of the CT and a decrease of the MCF and α-angle in the InTem analysis. Addition of fibrinogen, to correct for the dilutional effect, clearly recovered the ExTem and InTem ROTEM analysis. However, the InTem ROTEM analysis was not completely recovered by addition of heparin containing PCCs.

## Discussion

This is the first study investigating different commercially available PCCs in an *in vitro* hemodilution model. Despite the clear procoagulant effects of PPCs, the presence of heparin and antithrombin may provide PCC with some anticoagulant properties, as demonstrated in the current study. Using a panel of coagulation assays we were able to demonstrate the procoagulant effects of PCCs free or low dose of heparin, whereas the heparin containing PCCs behaved in an anticoagulant manner, depending on the degree of hemodilution.

Except for factor IX, the assessed coagulation factor composition of different PCCs was in accordance with the concentration provided by the manufactures. Moreover, procoagulant factors in the five different PCCs were comparable between each other as previously described by Kalina et al. [13]. In contrast, factor IX levels were lower as compared to the values on the label. One explanation for the observed lower factor IX levels may be the presence of heparin in the PCCs, which may influences the aPTT-based factor IX assay. This hypothesis is supported by the, although not complete, recovery of factor IX levels upon dilution of the PPC to heparin levels not influencing the aPTT based factor IX assay (data not shown). Three out of five PCCs did not report the presence of the anticoagulants protein C and S, while comparable levels were determined in all types of PCC. Moreover, the biochemical analysis from Kalina et al. indicated that Cofact does not contain protein C, whereas data from our analysis as well from the manufacturer demonstrated the presence of this anticoagulant protein in this PCC [13]. Heparin was present in four out of five PCCs with the highest levels in Octaplex 500. Remarkably, the presence of heparin was not indicated for prothromplex but the levels were comparable to those in Octaplex 500. Although both Octaplex 500 and PPSB-human SD/Nano 600 are from the same manufacturer, the heparin content in PPSB-human SD/Nano 600 is almost one third of that in Octaplex 500.

The balance between pro- and anticoagulant agents in PCC may theoretically determine the prothrombotic capacity of a given PCC. Before and around the mid-1990s, PCCs contained activated coagulation factors [19] and mostly lacked natural or artificial anticoagulant agents, which added to the risk of unwanted or excessive (activation of) coagulation upon administration. Currently available PCCs have shown to be safe in several studies in the treatment of oral anticoagulation reversal (vitamin K antagonists) [14], [20]. However, for the use of PCC in trauma induced coagulopathy the evidence for the safety and efficacy of PCC is very limited [5]–[7], [9], [16], [17]. To address this we applied two concentrations of Cofact in an animal model of blunt liver injury with coagulopathy [17]. Treatment with 35 IU PCC per kg was effective to reverse hemodilution and trauma induced coagulopathy both under normothermia and severe hypothermia [9], [17]. However, at a higher dose of 50 IU PCC per kg bodyweight 44% of the animals developed severe DIC with fibrin precipitation in lung capillaries and thrombemboli. *In vitro* plasma thrombin generation analyses revealed that these side effects could be contributed to a relatively high thrombin generating capacity, related to an overload in procoagulant factors and insufficient concentrations of anticoagulant agents (antithrombin, Protein C/S, or heparin). This observation suggests that a balance in pro- and anticoagulant agents may be an important determinant of the safety of PCC therapy.

As PCCs are only standardized according to factor IX levels it is difficult to assess the impact of available PCCs on overall coagulation assays. In the presented *in vitro* study all tested PCCs without (Cofact) or low concentration of heparin (Beriplex) exhibited a similar impact on plasma thrombin generation. The similar impact on thrombin generation of Cofact and Beriplex reinforces the idea that these PCCs are balanced with regard to their pro- and anticoagulatory properties. Due to the lack of evidence from prospective pre- and clinical studies no conclusions about the safety and efficacy of PCCs in the treatment of trauma induced coagulopathy can be drawn [16]. In contrast to PCCs with low concentrations of Heparin, PCCs containing higher concentrations of heparin (Prothromplex, Octaplex 500, PPSB-human SD/Nano 600) showed a lower impact on thrombin generation. Correspondingly, two *in vitro* investigation showed a dose depended increase in anticoagulation of PCCs containing high concentrations of heparin [21], [22].

### Conclusions

Overall these findings suggest the need for a balance between pro-and anticoagulants in PCC products. The question remains whether a heparin containing PCC would have the same unwanted prothrombotic side effect as observed for the non-heparin PCC. Thus there is an urgent need for further *in vivo* investigations to elaborate the safety and efficacy of PCCs to treat trauma-induced coagulopathy. As no point of care method to measure thrombin generation is currently available, further technical developments should focus on suitable and in-time measurements of thrombin generation.
